# Lipoprotein(a), family history, and incidence of premature ASCVD events in a pooled US cohort

**DOI:** 10.1016/j.ajpc.2025.101319

**Published:** 2025-10-03

**Authors:** Yihang Fan, Wenjun Fan, Xingdi Hu, Michael Y. Tsai, Ron C. Hoogeveen, Matthew J. Budoff, Nathan D. Wong

**Affiliations:** a Department of Epidemiology and Biostatistics, Joe C. Wen School of Population and Public Health, University of California, Irvine, USA; b Mary and Steve Wen Cardiovascular Division, School of Medicine, University of California, Irvine, USA; c Novartis Pharmaceutical Corporation, USA; d University of Minnesota, USA; e Baylor College of Medicine, USA; f Lundquist Institute, Harbor-UCLA Medical Center, USA

**Keywords:** Lipoprotein(a), Family history, Atherosclerotic cardiovascular disease

## Abstract

**Background:**

Lipoprotein(a) [Lp(a)] is an independent, genetic, and causal risk factor for atherosclerotic cardiovascular disease (ASCVD). There are limited data on its impact on premature ASCVD, including in diverse populations and with family history. We examined Lp(a) in relation to premature ASCVD (male aged <55, female aged <65 years) compared to later onset ASCVD, and differences by family history, sex, and race/ethnicity in a large, multi-ethnic U.S. cohort.

**Methods:**

We analyzed data from 27,756 individuals without prior ASCVD at baseline from a pooled cohort consisting of five U.S. prospective studies. Lp(a) levels were stratified by cohort-specific percentiles. Multivariable Cox regression assessed the association of Lp(a) with composite incident premature and non-premature ASCVD events by sex, race, and family history.

**Results:**

Among 5276 ASCVD events over a mean follow-up of 21.1 years, 773 (14.7 %) were premature ASCVD events. A higher proportion of women (65.2% vs. 38.3%) and Black individuals (45.8% vs. 27.7%) were observed in individuals with premature ASCVD compared to those with non-premature ASCVD events. For each 50 mg/dL increase in Lp(a), the risk of premature ASCVD increased by 30 % (HR: 1.30, 95% CI: 1.28–1.51), compared to a 24 % increase for non-premature ASCVD (HR: 1.24 [1.14–1.33]). Compared with Lp(a) levels <50th percentile, Lp(a) levels ≥ 90th percentile had adjusted HRs of 1.39 (1.10–1.75) and 1.39 (1.26–1.54) for premature and non-premature ASCVD events, respectively. We observed a trend for elevated Lp(a) levels predicting premature ASCVD events more strongly in those with a family history of ASCVD and in White individuals.

**Conclusion:**

Elevated Lp(a) is an important predictor of both premature and later onset ASCVD events.

Lipoprotein(a) [Lp(a)] is a genetic, independent, causal risk driver, for atherosclerotic cardiovascular disease (ASCVD).^1^ Elevated Lp(a) levels are largely hereditary and remain relatively stable throughout life, contributing to lifelong exposure if levels are high.^2^ Lp(a) is a well-established predictor of ASCVD events from large-scale epidemiologic studies, Mendelian randomization, and genome-wide association studies.^1^^,^[3], [4], [5]

Premature ASCVD is typically defined by an event occurring before age 55 years in men and 65 years in women​.^6^ Individuals who develop ASCVD at younger ages often have a high lifetime disease burden and may have unrecognized genetic or familial risk factors, underscoring the need for earlier assessment of ASCVD risk.^7^ Currently, most evidence comes from middle-aged or older populations, and there are limited data that have examined the impact of Lp(a) in younger individuals. The Cardiovascular Risk in Young Finns Study found that Lp(a) levels ≥ 30 mg/dL, measured in childhood or young adulthood, were associated with a 2-fold greater risk of developing future ASCVD events at a median age of 47 years.^8^ However, both conventional and Mendelian randomization analyses from the same study found no association between Lp(a) and the progression of brachial flow-mediated dilatation or carotid atherosclerosis.^9^

While elevated Lp(a) levels consistently predict overall ASCVD events, whether it predicts premature ASCVD similarly compared to later-onset events remains uncertain.^4^^,^^5^^,^^10^ In addition, the impact of family history on the association between Lp(a) and premature ASCVD has not been well established in large and diverse cohorts. Our previous analysis pooling 5 major US cohort studies demonstrated that higher Lp(a) levels were independently associated with increased ASCVD risk, with a particularly strong association at the highest percentiles of Lp(a), especially in those with diabetes.^4^ This study will build on those findings by assessing whether Lp(a) more strongly predicts premature ASCVD compared to later-onset events, and how a family history of ASCVD, sex or race/ethnicity may modify these relationships.

## Methods

We included participants from five major prospective cardiovascular studies: Multi-Ethnic Study of Atherosclerosis (MESA), Coronary Artery Risk Development in Young Adults (CARDIA), Jackson Heart Study (JHS), Framingham Heart Study Offspring Cohort (FHS-OS), and the Atherosclerosis Risk in Communities (ARIC) (Central Illustration). Details on study design, cohort recruitment, data collection, and laboratory assessments have been previously published.^4^ In brief, participants without prior ASCVD at baseline were included, with follow-up for incident premature and non-premature ASCVD events. The analysis of data for this paper was approved by the University of California–Irvine Institutional Review Board as protocol #2020–6390.

**Central Illustration:**
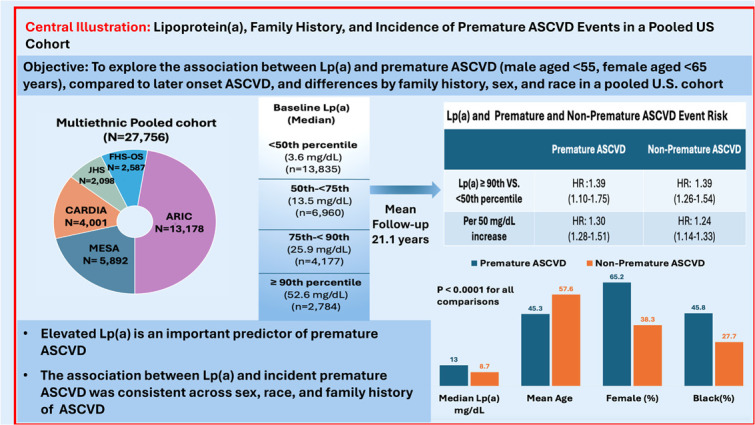
Lipoprotein(a), Family History, and Incidence of Premature ASCVD Events in a Pooled US Cohort.

Lp(a) concentrations were measured using turbidimetric immunoassay, ELISA, or Diasorin nephelometric assay across the included cohorts. Measurements were obtained during specific examination periods: MESA (examination 1, 2000–2002), CARDIA (examination 3, 1990–1991), JHS (examination 1, 2000–2004), FHS (examination 3, 1983–1987), and ARIC (examination 1, 1987–1989). To account for variability in absolute Lp(a) levels across cohorts and assay types, we categorized Lp(a) within each cohort into four percentile-based groups: <50th percentile, 50th–<75th percentile,75th–<90th percentile, and ≥90th percentile, followed by pooling of these percentile groupings across cohorts. This is appropriate to minimize potential biases from differences in absolute Lp(a) values observed between cohorts due to differences in assays used. In addition, Lp(a) was also analyzed as a continuous variable based on a 50 mg/dL increment.^11^^,^^12^

ASCVD events were defined as a composite of nonfatal MI and stroke, revascularization, and coronary heart disease (CHD) death and were ascertained through adjudicated medical record reviews, death certificates, and hospitalization records, with cohort-specific endpoint committees verifying events using standardized adjudication protocols. Follow-up for ASCVD events was extended through 2014 (JHS), 2016 (CARDIA, ARIC), 2017 (MESA), and 2019 (FHS). In this study, we further stratified ASCVD events into premature and non-premature categories. Premature ASCVD was defined as an event occurring before age 55 years in men and before age 65 years in women, while non-premature ASCVD was defined as events occurring at or beyond these age thresholds.

We compared the baseline characteristics among premature and non-premature ASCVD cohorts using the Chi-square test for categorical variables and analysis of variance for continuous variables. Covariates included Lp(a) (per SD), demographic factors (age, sex, race/ethnicity, education level), study cohort, and traditional cardiovascular risk factors (blood pressure, body mass index, lipid parameters, smoking status, diabetes, and family history of ASCVD). Family history of ASCVD was defined based on self-reported parental or sibling history of myocardial infarction or stroke, using cohort-specific variables across the five pooled studies. Incidence rates of premature and non-premature ASCVD events were calculated per 1000 person-years across Lp(a) categories. Cox proportional hazards regression was used to assess the association between Lp(a) percentile groups (with <50th percentile as the reference) and the risk of premature and non-premature ASCVD in separate models. Additionally, Cox models evaluated how an Lp(a) increment of 50 mg/dL affected both premature and non-premature ASCVD risk. The follow-up time was defined from the examination at which Lp(a) was measured to the first qualifying ASCVD event or the most recent available follow-up. Analyses were adjusted for age, sex, race/ethnicity, education level, study cohort, systolic and diastolic blood pressure, body mass index, LDL-C, HDL-C, triglycerides, smoking status, diabetes, family history of ASCVD, and cholesterol medication. Additional stratified analyses were conducted by sex, race (Black, White, and Other), and family history of ASCVD. Interaction p-values were provided to assess differences in associations across strata.

## Results

We included 27,756 persons without previous ASCVD at baseline, of which 5,276 experienced incident ASCVD events during the follow-up period, with 773 being premature and 4,503 being non-premature ASCVD events. The mean age was 51.2 ± 12.4 years, with significant differences between the premature ASCVD group (45.3 ± 9.9 years) and the non-premature ASCVD group (57.6 ± 8.3 years) (*p* < 0.0001). For females, mean ages were 48.1 ± 9.2 and 58.2 ± 8.2, and males 39.9 ± 8.9 and 57.2 ± 8.4 for those with premature and non-premature ASCVD events, respectively (*p* < 0.0001). Overall, 55.0 % of participants were female, with a higher proportion in the premature ASCVD events (65.2 %) compared to the non-premature ASCVD events (38.3 %). Racial distribution varied across groups, with Black participants representing a higher proportion of patients with premature ASCVD events (45.8 %) compared to those with non-premature ASCVD cases (27.7 %) (*p* < 0.0001) (Table 1 and Central Illustration). The mean follow-up time was 21.1 ± 9.9 years for overall ASCVD events. Median Lp(a) levels were higher in the premature ASCVD group (13.0 mg/dL) compared to the non-premature ASCVD group (8.7 mg/dL) (*p* < 0.0001). Additional baseline characteristics, including BMI, LDL-C, smoking status, and medication use, are presented in Table 1.

**Table 1:** Demographic Characteristics in ASCVD patients in the Pooled Cohort by Premature ASCVD and Non-Premature ASCVD.

	Overall (n=27756)	Non-ASCVD (n=22480)	Overall ASCVD (n=5276)	Event types		
				Premature (n=773)	Non-Premature (n=4503)	P-Value
**Sex**						<0.0001
Female	15270 (55.0%)	13043 (58.0%)	2227 (42.2%)	504 (65.2%)	1723 (38.3%)	
Male	12486 (45.0%)	9437 (44.0%)	3049 (57.8%)	269 (34.8%)	2780 (61.7%)	
**Race**						<0.0001
White	15870 (57.2%)	12473 (55.5%)	3397 (64.4%)	395 (51.1%)	3002 (66.7%)	
Asian	681 (2.5%)	605 (2.7%)	76 (1.4%)	4 (0.5%)	72 (1.6%)	
Black	9859 (35.6%)	8295 (36.9%)	1600 (30.3%)	354 (45.8%)	1246 (27.7%)	
Hispanic	1310 (4.7%)	1107 (4.9%)	203 (3.9%)	20 (2.6%)	183 (4.1%)	
**Age, y**	51.2±12.4	50.2±12.7	55.8±9.6	45.3±9.9	57.6±8.3	<0.0001
**Education level**						0.0001
High school or less	15755 (56.8%)	12259 (54.5%)	3496 (66.3%)	482 (62.4%)	3014 (66.9%)	
College	8551 (30.8%)	7370 (32.8%)	1181 (22.4%)	217 (28.1%)	964 (21.4%)	
Graduate school	3450 (12.4%)	2851 (12.7%)	599 (11.4%)	74 (9.6%)	525 (11.7%)	
**Median lp(a) (mg/dL)**	10.8	11.0	9.2	13.0	8.7	<0.0001
**Lp(a) (mg/dL)**	20.5±27.2	21.2±27.7	17.5±24.6	23.1±31.8	16.5±23.0	<0.0001
**Female**	22.1±28.8	22.5±28.9	19.8±27.8	24.7±34.4	18.4±25.4	<0.0001
**Male**	18.5±24.9	19.4±25.7	15.7±21.8	20.1±26.0	15.3±21.3	<0.0001
**White**	12.1±17.2	12.2±17.5	11.8±16.2	15.1±20.4	11.4±15.5	<0.0001
**Asian**	22.6±23.8	22.2±23.4	25.9±26.8	38.7±23.8	25.2±26.9	<0.0001
**Black**	32.8±34.0	33.8±34.1	27.6±32.6	31.6±39.3	26.5±30.4	0.0006
**Hispanic**	27.0±31.6	26.6±31.0	29.0±34.6	27.9±28.6	29.1±35.2	0.2987
**With Family history of ASCVD**	21.8±29.4	22.8±30.0	18.6±26.9	26.3±35.2	17.2±24.9	<0.0001
**W/O Family history of ASCVD**	19.4±25.3	20.0±25.8	16.3±21.9	19.4±26.8	15.8±20.9	0.0004
**Systolic blood pressure (mm Hg)**	120.7±18.9	119.2±18.3	127.4±19.9	125.9±22.2	127.67±19.4	0.0228
**Diastolic blood pressure (mm Hg)**	73.4±10.8	72.8±10.5	76.0±11.6	77.3±13.3	75.7±11.3	0.0003
**Body mass index (kg/m^2^)**	27.8±5.8	27.6±5.8	28.4±5.3	29.3±6.6	28.3±5.1	<0.0001
**Glucose (mg/dL)**	99.9±33.6	97.0±29.1	112.0±46.4	114.1±61.0	111.6±43.4	0.1744
**Total cholesterol (mg/dL)**	203.5±41.3	200.5±40.3	216.1±43.3	214.1±45.8	216.5±42.8	0.1588
**LDL-C (mg/dL)**	128.1±37.6	125.2±36.5	140.4±39.3	138.3±42.2	140.8±38.8	0.1241
**HDL-C (mg/dL)**	51.8±15.7	52.9±15.8	47.3±14.6	47.8±14.3	47.1±14.7	0.3000
**Triglycerides (mg/dL)**	119.7±89.7	113.7±86.1	145.3±99.6	143.1±114.1	145.7±96.9	0.4963
**Diagnosed diabetes mellitus**	2100 (7.6%)	1376 (6.1%)	724 (13.7%)	129 (16.7%)	595 (13.2%)	0.0095
**Current smoker**	6795 (24.5%)	5319 (23.6%)	1476 (28.0 %)	375 (48.5%)	1101 (24.5%)	<0.0001
**Hypertension medication**	6852 (24.7%)	4961 (22.1%)	1891 (35.5%)	259 (33.5%)	1632 (36.2%)	0.1427
**Cholesterol medication**	1213 (4.4%)	939 (4.2%)	274 (5.2%)	25 (3.2%)	249 (5.5%)	0.0079
**Family history of ASCVD**	12116 (43.7%)	9415 (41.9%)	2701 (51.2%)	414 (53.6%)	2287 (50.8%)	0.1547

Values are mean ± SD or n (%), unless otherwise indicated.

ASCVD :atherosclerotic cardiovascular disease; HDL-C : high-density lipoprotein cholesterol; LDL-C : low-density lipoprotein-cholesterol; Lp(a) : lipoprotein(a).

**Fig. 1:**
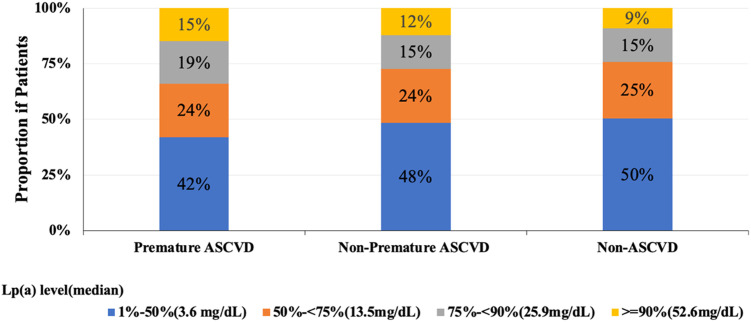
Distribution of Lipoprotein(a) Levels According to Premature, Non-Premature, and Non-ASCVD Events *p* < 0.01 comparing premature vs. non-premature and *p* < 0.0001 comparing premature or non-premature with non-ASCVD events.

Fig. 1 shows the distribution of Lp(a) levels across premature ASCVD, non-premature ASCVD, and non-ASCVD groups. Median Lp(a) levels in the pooled cohort were 3.6 mg/dL for <50th percentile, 13.5 mg/dL for the 50–<75th, 25.9 mg/dL for the 75–<90th, and 52.6 mg/dL for the ≥90th percentile group. Elevated Lp(a) (≥90th percentile) was found in 15 % of those with premature ASCVD compared to 12 % in those with non-premature ASCVD and 9 % in those without ASCVD. Conversely, lower Lp(a) levels (1 %−50 %) were most common in non-ASCVD (50 %) and least in premature ASCVD (42 %). Lp(a) levels were significantly different across all pairwise group comparisons: premature vs. non-premature ASCVD (*p* = 0.0028), premature ASCVD vs. non-ASCVD (*p* < 0.0001), and non-premature ASCVD vs. non-ASCVD (*p* < 0.0001).

Table 2 and the Central Illustration present the multivariable Cox regression results for relationship between Lp(a) and premature and non-premature ASCVD events. Overall, higher Lp(a) levels were associated with increased ASCVD risk, with a trend towards a stronger effect in premature ASCVD. A 50 mg/dL increase in Lp(a) was significantly associated with a 30 % higher risk of premature ASCVD (HR: 1.30[1.28–1.51, *p* = 0.0004]) and a 24 % higher risk of non-premature ASCVD (HR: 1.24 [1.14–1.33], *p* < 0.0001). Individuals with Lp(a) of ≥90th percentile had a 39 % increased risk of premature ASCVD compared to those in the < 50th percentile (HR: 1.39[1.10–1.75], *p* = 0.0052), while those in the 75th-90th percentile had a 23 % increased risk (HR: 1.23; [1.00–1.51], *p* = 0.0466). Similarly, individuals in the highest Lp(a) category (≥90th percentile) had a 39 % increased risk of non-premature ASCVD (HR: 1.39[1.26–1.54], *p* < 0.0001), while those in the 75th-90th percentile had a 13 % increased risk (HR: 1.13[1.04–1.24], *p* = 0.0009).

**Table 2:** Lp(a) and Premature and Non-Premature ASCVD events risk.

Premature (men<55 yo, women<65 yo)				
	Event/Sample	Incidence Rate (per 1000 person-years)	Unadjusted HR (95% CI)	Adjusted HR (95% CI)
Overall (per 50 mg/dL)	773/27756	1.32	1.35 (1.21-1.51) ***	1.30 (1.28-1.51) **
Lpa(Category)				
<50%tile	326/13835	1.11	Reference	Reference
50-<75%tile	187/6960	1.27	1.15 (0.96-1.38)	0.98 (0.81-1.18)
75-<90%tile	147/4177	1.69	1.52 (1.25-1.85) ***	1.23 (1.00-1.51) *
>=90%tile	113/2784	1.98	1.77 (1.43-2.19) ***	1.39 (1.10-1.75) **

Non-Premature (men>=55 yo, women>-65 yo)				
	Event/Sample	Incidence Rate (per 1000 person-years)	Unadjusted HR (95% CI)	Adjusted HR (95% CI)
Overall (per 50 mg/dL)	4503/27756	7.70	1.12 (1.05-1.20) **	1.24 (1.14-1.33)***
Lpa(Category)				
<50%tile	2177/13835	7.40	Reference	Reference
50-<75%tile	1088/6960	7.41	1.00 (0.93-1.08)	1.06 (0.98-1.14)
75-<90%tile	692/4177	7.98	1.09 (1.00-1.19) *	1.13 (1.04-1.24) **
>=90%tile	546/2784	9.58	1.33 (1.21-1.46) ***	1.39 (1.26-1.54) ***

*P < 0.05, **P < 0.01; and ***P < 0.0001

Covariates in multivariable Cox regression models for adjusted HRs include age, sex, race, education level, study cohort, systolic and diastolic blood pressure, body mass index, diabetes, low-density lipoprotein cholesterol, high-density lipoprotein cholesterol, triglycerides, diabetes, smoking status, family history of cardiovascular disease, and cholesterol medication

**Fig. 2:**
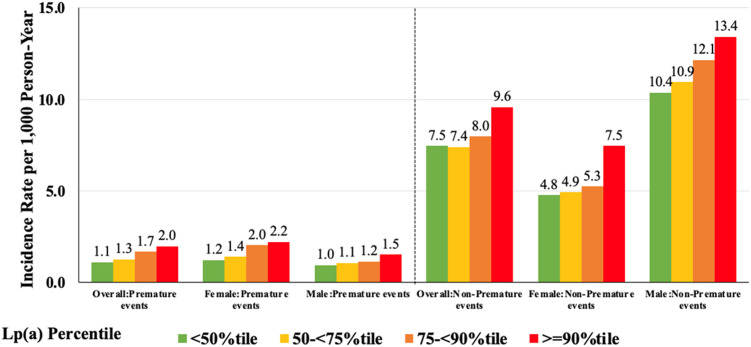
Premature and Non-Premature ASCVD Event Rates Per 1000 Person Years by Lipoprotein(a) Percentile Group and Sex.

Table 3 presents the sex-stratified associations between Lp(a) and premature and non-premature ASCVD. The association between a 50mg/dL increase in Lp(a) level and premature ASCVD event risk was higher in females (HR: 1.36[1.14–1.61], *p* < 0.01), but no significant association was observed in males (HR: 1.25[0.94–1.65]). Compared to the lowest < 50th percentile, females with Lp(a) levels in the ≥90th percentile had a 39 % increased risk of premature ASCVD, while the association was slightly stronger in males but not statistically significant (HR:1.41 [0.93–2.14]). Interaction tests showed no significant difference between females and males for premature ASCVD (*p* = 0.51) and non-premature ASCVD (*p* = 0.73). Fig. 2 illustrates the incidence rates of ASCVD events per 1000 person-years, stratified by Lp(a) percentile and ASCVD type (premature vs non-premature) in both sexes. A clear gradient is observed, with higher Lp(a) levels associated with increased ASCVD risk. Among individuals with premature ASCVD, event rates are highest in females at the ≥90th percentile (2.2 per 1000 person-years), nearly 50 % greater than the rate observed in males (1.5 per 1000 person-years at the same percentile). However, these differences were not statistically significant across any Lp(a) percentile in the premature group. In contrast, in non-premature ASCVD, men with Lp(a) in the ≥90th percentile show the highest incidence rate (13.4 per 1000 person-years), and event rates in males were consistently higher than females across all Lp(a) categories (*p* < 0.0001 for each quantile comparison).

**Table 3:** Lp(a) and Premature and Non-Premature ASCVD events Risk by Sex.

	Female (n=15270)		Male (n=12486)	
Premature ASCVD Events				
	Unadjusted HR (95% CI)	Adjusted HR (95% CI)	Unadjusted HR (95% CI)	Adjusted HR (95% CI)
Overall (per 50 mg/dL)	1.32 (1.16-1.50) ***	1.36 (1.14-1.61) **	1.34 (1.08-1.66) *	1.25 (0.94-1.65)
Lpa(Category)				
<50%tile	Reference	Reference	Reference	Reference
50-<75%tile	1.15 (0.91-1.44)	0.95 (0.75-1.20)	1.11 (0.83-1.50)	1.10 (0.81-1.49)
75-<90%tile	1.65 (1.30-2.08) ***	1.31 (1.02-1.69) *	1.20 (0.84-1.71)	1.04 (0.72-1.51)
>=90%tile	1.77 (1.37-2.30) ***	1.39 (1.06-1.84) *	1.59 (1.08-2.35) *	1.41 (0.93-2.14)
**Non-Premature ASCVD Events**				
Overall (per 50 mg/dL)	1.32 (1.20-1.46) ***	1.33 (1.18-1.49) ***	1.10 (1.00-1.20) *	1.10 (1.08-1.33) **
Lpa(Category)				
<50%tile	Reference	Reference	Reference	Reference
50-<75%tile	1.03 (0.92-1.17)	1.03 (0.91-1.16)	1.06 (0.97-1.16)	1.07 (0.98-1.18)
75-<90%tile	1.12 (0.98-1.29)	1.07 (0.92-1.23)	1.18 (1.06-1.32) **	1.19 (1.06-1.33) **
>=90%tile	1.64 (1.42-1.88) ***	1.44 (1.25-1.67) ***	1.31 (1.15-1.49) ***	1.35 (1.18-1.55) ***

*P < 0.05, **P < 0.01; and ***P < 0.0001

Covariates for adjusted HRs include age, sex, race, education level, study cohort, systolic and diastolic blood pressure, body mass index, diabetes, low-density lipoprotein cholesterol, high-density lipoprotein cholesterol, triglycerides, diabetes, smoking status, family history of cardiovascular disease, and cholesterol medication

Interaction P = 0.51 between sex for premature ASCVD Events

Interaction P = 0.73 between sex for Non-premature ASCVD Events

**Fig. 3:**
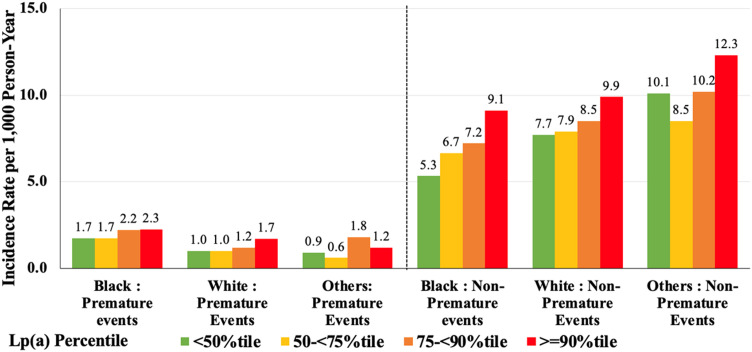
Premature and Non-Premature ASCVD Event Rates Per 1000 Person Years by Lipoprotein(a) Percentile Group and Race.

Table 4 and Fig. 3 present the association between Lp(a) and premature and non-premature ASCVD stratified by race. Among Black individuals, those in the highest Lp(a) category (≥90th percentile) had a 52 % increased risk of non-premature ASCVD (HR: 1.52 [1.27–1.81], *p* < 0.0001) compared to those < 50th percentile, but no significant association was observed for premature ASCVD. In contrast, among white individuals, the relationship between Lp(a) and ASCVD was more pronounced, with a 74 % increased risk of premature ASCVD per 50 mg/dL increase (HR: 1.74[1.33–2.27], *p* < 0.0001) and a 29 % increased risk of non-premature ASCVD (HR: 1.29; [1.14–1.46], *p* < 0.0001). Compared to the <50th percentile groups, the ≥90th percentile group had an almost 2 times higher risk of premature ASCVD (HR: 1.89[1.36–2.63], *p* < 0.0001) and a 40 % increased risk of non-premature ASCVD (HR: 1.40; [1.23–1.60], *p* < 0.0001). As shown in Fig. 3, ASCVD incidence rates were higher in White and Other race individuals for non-premature ASCVD. In Black,White and Other individuals, the incidence rates for premature events were similar. In contrast, for non-premature ASCVD, the trend was more obvious in Black individuals, with a clear increase in event rates as Lp(a) percentile increased. White individuals with Lp(a) ≥90th percentile had an event rate of 9.9 per 1000 person-years for non-premature ASCVD, compared with 9.1 per 1000 person-years in Black individuals. Interaction tests showed no significant difference between different race individuals for premature ASCVD (*p* = 0.23) and non-premature ASCVD (*p* = 0.71).

**Table 4:** Lp(a) and Premature and Non-Premature ASCVD events Risk by Race.

	Black (n=9885)		White (n=15870)		Others (n=1991)	
Premature ASCVD Events						
	Unadjusted HR (95% CI)	Adjusted HR (95% CI)	Unadjusted HR (95% CI)	Adjusted HR (95% CI)	Unadjusted HR (95% CI)	Adjusted HR (95% CI)
Overall (per 50 mg/dL)	1.13 (0.97-1.33)	1.20 (0.99-1.45)	1.55 (1.24-1.93) ***	1.74 (1.33-2.27) ***	1.23 (0.69-2.19)	1.24 (0.72-2.14)
Lpa(Category)						
<50%tile	Reference	Reference	Reference	Reference	Reference	Reference
50-<75%tile	1.00 (0.76-1.32)	0.95 (0.72-1.27)	1.04 (0.80-1.34)	0.98 (0.75-1.27)	0.62 (0.18-2.16)	1.56 (0.16-2.01)
75-<90%tile	1.28 (0.96-1.71)	1.05 (0.77-1.42)	1.32 (0.99-1.78)	1.33 (0.99-1.79)	2.01 (0.73-5.59)	1.71 (0.59-4.92)
>=90%tile	1.29 (0.94-1.76)	1.07 (0.76-1.49)	2.00 (1.45-2.75) ***	1.89 (1.36-2.63) ***	1.45 (0.33-6.39)	2.05 (0.43-9.77)
**Non-Premature ASCVD Events**						
Overall (per 50 mg/dL)	1.12 (1.01-1.23) *	1.33 (1.19-1.49) ***	1.08 (0.96-1.21)	1.29 (1.14-1.46) ***	1.37 (0.94-1.38)	1.95 (0.97-1.47)
Lpa(Category)						
<50%tile	Reference	Reference	Reference	Reference	Reference	Reference
50-<75%tile	1.25 (1.07-1.45) **	1.17 (1.00-1.37)	0.98 (0.90-1.08)	1.05 (0.96-1.15)	0.84 (0.61-1.17)	0.89 (0.64-1.24)
75-<90%tile	1.35 (1.14-1.58) **	1.15 (0.97-1.37)	1.09 (0.97-1.21)	1.20 (1.08-1.35) **	1.03 (0.69-1.52)	1.08 (0.72-1.62)
>=90%tile	1.73 (1.46-2.04) ***	1.52 (1.27-1.81) ***	1.25 (1.10-1.42) **	1.40 (1.23-1.60) ***	1.24 (0.78-1.97)	1.38 (0.84-2.25)

*P < 0.05, **P < 0.01; and ***P < 0.0001

Others include Hispanic and Asian

Covariates for adjusted HRs include age, sex, race, education level, study cohort, systolic and diastolic blood pressure, body mass index, diabetes, low-density lipoprotein cholesterol, high-density lipoprotein cholesterol, triglycerides, diabetes, smoking status, family history of cardiovascular disease, and cholesterol medication

Interaction P = 0.23 between races for premature ASCVD Events

Interaction P = 0.71 between races for Non-premature ASCVD Events

**Fig. 4:**
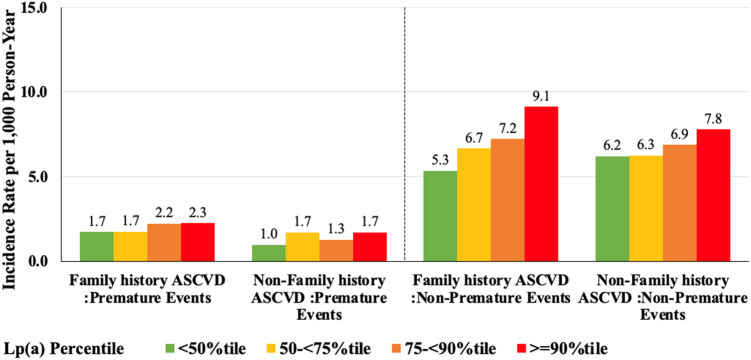
Premature and Non-Premature ASCVD Event Rates Per 1000 Person Years by Lipoprotein(a) Percentile Group and Presence of Family History of ASCVD.

Table 5 and Fig. 4 show the relationship between Lp(a) and ASCVD, stratified by family history. Among individuals with a family history of ASCVD, Lp(a) was significantly associated with both premature (HR: 1.43 per 50 mg/dL increase; 95 % CI: 1.19–1.71, *p* < 0.0001) and non-premature ASCVD (HR: 1.35 per 50 mg/dL increase [1.22–1.50], *p* < 0.0001). Compared to those in the <50th percentile, individuals with a family history of ASCVD in the 75-<90th and ≥90th percentiles had a 47 % and 61 % higher risk of premature ASCVD, and a 18 % and 52 % higher risk of non-premature ASCVD, respectively. Among those without a family history, associations were not statistically significant for premature ASCVD (HR: 1.14 per 50 mg/dL increase; 95 % CI: 0.89–1.47, *p* > 0.05) and non-premature ASCVD (HR: 1.12 per 50 mg/dL increase; 95 % CI: 1.00–1.26, *p* > 0.05). Individuals in the ≥90th percentile had a 27 % increased risk of non-premature ASCVD. Interaction testing between Lp(a) and family history showed no statistically significant effect modification (premature ASCVD: *p* = 0.07; non-premature ASCVD: *p* = 0.40). The borderline *p* = 0.07 for premature ASCVD should be interpreted cautiously and may warrant follow-up in larger analyses. Fig. 4 shows that individuals with a family history of ASCVD had higher rates of premature ASCVD across all percentiles than those without a family history of ASCVD. For non-premature ASCVD, those with a family history had higher incidence rates than those without, except in the <50th percentile group. In the ≥90th percentile, the rate of non-premature ASCVD was highest among those with a family history of ASCVD (9.1 per 1000 person-years) compared to those without (7.8 per 1000 person-years).

**Table 5:** Lp(a) and Premature and Non-Premature ASCVD events Risk by Family history of ASCVD.

	With Family history of ASCVD (n=12116)		Without Family history of ASCVD (n=15640)	
Premature ASCVD Events				
	Unadjusted HR (95% CI)	Adjusted HR (95% CI)	Unadjusted HR (95% CI)	Adjusted HR (95% CI)
Overall (per 50 mg/dL)	1.40 (1.23-1.59) ***	1.43 (1.19-1.71) ***	1.18 (0.97-1.45)	1.14 (0.89-1.47)
Lpa(Category)				
<50%tile	Reference	Reference	Reference	Reference
50-<75%tile	1.06 (0.82-1.38)	0.91 (0.70-1.19)	1.24 (0.96-1.60)	1.05 (0.81-1.37)
75-<90%tile	1.74 (1.35-2.25)	1.47 (1.12-1.93) **	1.26 (0.93-1.70)	0.97 (0.70-1.33)
>=90%tile	1.93 (1.46-2.56)	1.61 (1.19-2.18) **	1.54 (1.10-2.14)	1.16 (0.81-1.64)
**Non-Premature ASCVD Events**				
Overall (per 50 mg/dL)	1.15 (1.06-1.26) **	1.35 (1.22-1.50) ***	1.07 (0.97-1.19)	1.12 (1.00-1.26)
Lpa(Category)				
<50%tile	Reference	Reference	Reference	Reference
50-<75%tile	1.01 (0.91-1.12)	1.08 (0.97-1.20)	1.00 (0.90-1.11)	1.04 (0.94-1.16)
75-<90%tile	1.05 (0.93-1.19)	1.18 (1.04-1.33) *	1.13 (1.00-1.27)	1.10 (0.97-1.25)
>=90%tile	1.32 (1.16-1.50)	1.52 (1.33-1.74) ***	1.30 (1.13-1.49) **	1.27 (1.10-1.47)**

*P < 0.05, **P < 0.01; and ***P < 0.0001

Covariates for adjusted HRs include age, sex, race, education level, study cohort, systolic and diastolic blood pressure, body mass index, diabetes, low-density lipoprotein cholesterol, high-density lipoprotein cholesterol, triglycerides, diabetes, smoking status, family history of cardiovascular disease, and cholesterol medication

Interaction P = 0.07 between with and without family history groups for premature ASCVD Events

Interaction P = 0.40 between with and without family history groups for Non-premature ASCVD Events

## Discussion

Our study is the largest and most diverse U.S.-based cohort analysis examining the relationship between Lp(a) and premature/non-premature ASCVD in primary prevention, with significant representation of female and Black participants. Individuals with premature ASCVD had higher median Lp(a) levels and a greater prevalence of elevated Lp(a) compared to those with non-premature ASCVD. The premature ASCVD group also had a higher proportion of female and Black participants compared to the non-premature ASCVD group. We found that elevated Lp(a) was similarly associated with both premature and non-premature ASCVD events, but with a trend towards a stronger relationship observed for the prediction of premature ASCVD events in those with versus without a family history of ASCVD, as well as in White compared to Black individuals.

Our previous pooled cohort analysis demonstrated a strong association between elevated Lp(a) and ASCVD risk, and the findings from this study provide additional insights into Lp(a) as a strong predictor for premature ASCVD events. In the prior study, compared with Lp(a) levels <50th percentile, Lp(a) levels in the 50th to <75th, 75th to <90th, and ≥90th percentiles had adjusted HRs of 1.06 (95 % CI: 0.99–1.14), 1.18 (95 % CI: 1.09–1.28), and 1.46 (95 % CI: 1.33–1.59), respectively for ASCVD events.^4^ Our findings are consistent, both for the prediction of premature and non-premature ASCVD events. Notably, a recent meta-analysis including 100,540 participants, with a mean age range of 35–62 years, found that elevated Lp(a) was associated with about a two-fold increased odds of composite ASCVD in this age group, with significant findings in both men (OR: 2.31, 95 % CI: 1.81–2.97) and women (OR: 2.34, 95 % CI: 1.53–3.58), reinforcing that Lp(a) is a robust risk factor in the young.^13^ Consistent with this meta-analysis, our study found a trend towards a stronger association between elevated Lp(a) and premature ASCVD compared to non-premature ASCVD and especially in those with a family history of ASCVD and in White persons. Our results provide new insights from a diverse U.S. cohort, supporting this conclusion by examining the differential impact of Lp(a) across sex, race, and family history of ASCVD, which has been an area of ongoing debate.

We observed sex and race/ethnicity differences in the composition of premature vs. non-premature ASCVD events, and the association between Lp(a) and premature versus later onset ASCVD outcomes were stronger in females. Previous studies have shown that postmenopausal women tend to have elevated Lp(a) levels, with menopausal status being the strongest predictor of Lp(a) increase.[14], [15], [16] This is consistent with our findings, where we observed that, compared to the lowest Lp(a) category, females in the elevated Lp(a) ≥90th percentile have a slightly higher risk of developing later-onset ASCVD than premature ASCVD. Contrary to our findings, a recent analysis from the Women’s Health Study found that Lp(a) was only associated with premature CHD (occurring by age 65) but not with later-onset CHD.^17^ Regarding race, Black individuals made up a larger proportion of the premature ASCVD group (46 %) compared to the non-premature group (28 %), but the association with premature ASCVD events was stronger in White individuals. This discrepancy could be explained by the fact that Black individuals more often have smaller apo(a) isoforms, so mass-based assays (mg/dL) can obscure particle-level risk, and by a greater burden of cardiovascular risk factors in Black populations, particularly hypertension, which contributes to premature events.^18^^,^^19^ Consistent with findings from the ARIC Study comparing African American and Caucasians population,^19^ elevated Lp(a) was associated with premature ASCVD in White populations, and the interaction test supported the role of Lp(a) in predicting premature ASCVD regardless of race.

In a recent multicohort analysis of MESA and ARIC, individuals with ≥2 first-degree relatives with premature ASCVD had a higher prevalence of elevated Lp(a) than those without a family history.^20^Another analysis using an ARIC study demonstrated that both family history of CVD and elevated Lp(a) independently predicted future ASCVD risk, with the highest risks observed in individuals with both factors (HR: 1.43, 95 % CI: 1.27–1.62). However, while no significant interaction between Lp(a) and family history was noted (*p* = 0.75), indicating that Lp(a) did not serve as a stronger predictor of ASCVD events in those with a family history.^21^ Similarly, a recent Chinese study of individuals with chronic coronary syndrome reported the highest major adverse cardiac event risk in those with both a family history of CHD and elevated Lp(a), compared to those with either or neither factor.^22^ Our findings show a trend towards a stronger association between elevated Lp(a) and premature ASCVD among those with family history of ASCVD.

Our study has several strengths and limitations. A key strength of our study is the inclusion of a multi-ethnic cohort with participants from diverse backgrounds, allowing for a comprehensive analysis of Lp(a) as a predictor of ASCVD events across diverse groups (although we had an insufficient number of events to assess our relationships in Asian and Hispanic persons). Additionally, the large sample size with long-term follow-up provided robust statistical power to assess the association between Lp(a) levels and both premature and non-premature ASCVD events. Despite these strengths, several limitations should be noted. First, the observational design precludes causal inference and residual confounding from unmeasured factors, such as inflammatory markers or Lp(a) isoform size, which may still affect the results. Second, Lp(a) measurements were performed at baseline using different assays across the different cohorts. While we mitigated calibration differences by analyzing Lp(a) in cohort-specific percentiles as well as adjusting for any cohort differences, minor misclassification may still have occurred. Moreover, while we stratified our results by family history of ASCVD, its high prevalence (44 % overall) makes the measure nonspecific and likely dilutes subgroup differences; in addition, we were not able to specifically identify across all cohorts whether the individual had a premature family history of ASCVD. Finally, the definition of premature ASCVD is widely used but sex-specific, which could confound direct sex comparisons. We conducted sex-stratified analyses and found consistent effects of Lp(a), but this age definition remains a nuance to interpret. Overall, the consistency of our findings with known biological mechanisms and other studies provides confidence in the validity of the observed associations.

Our analysis of a multi-ethnic pooled cohort study further highlights the strong association between elevated Lp(a) levels for both premature and later onset ASCVD events. We observed a trend towards stronger prediction of premature events, especially in those with a family history of ASCVD and in White persons. Our findings may inform the design and feasibility of future clinical trials targeting Lp(a) to reduce ASCVD risk at an early age. Moreover, consistent with US National Lipid Association^12^, our results support integrating Lp(a) screening into primary prevention strategies among all adults without preferential emphasis on any single subgroup. Although relative associations were stronger for premature events, their absolute event rates (and absolute risk differences across Lp(a) groups) were much lower; clinically, this would suggest higher numbers needed to treat for prevention of premature compared to later onset events for current and potential future therapies for Lp(a) lowering. To confirm and extend these findings, further studies in large-scale, real-world cohorts, including diverse ethnic groups and clinical settings, are needed. This would provide valuable insights into the clinical practice of Lp(a) screening and its role in preventing ASCVD across different populations

## Funding

This project was funded by a research contract from Novartis to the University of California, Irvine.

## Disclosures

Dr. Wong receives research support through the University of California, Irvine from Amgen and Novartis and is a consultant and speaker for Novartis and consultant for Ionis. Dr. Hoogeveen has received research support through Baylor College of Medicine, Houston from Denka Seiken and is a consultant for Denka Seiken. Dr. Hu is an employee of Novartis Pharmaceuticals Corporation. The remaining authors have no relevant disclosures.

## CRediT authorship contribution statement

**Yihang Fan:** Writing – review & editing, Writing – original draft, Software, Methodology, Formal analysis. **Wenjun Fan:** Writing – review & editing, Methodology, Formal analysis, Data curation. **Xingdi Hu:** Writing – review & editing, Project administration, Methodology, Conceptualization. **Michael Y. Tsai:** Writing – review & editing, Data curation. **Ron C. Hoogeveen:** Writing – review & editing. **Matthew J. Budoff:** Writing – review & editing, Data curation. **Nathan D. Wong:** Writing – review & editing, Project administration, Methodology, Investigation, Funding acquisition, Conceptualization.

## Declaration of competing interest

Dr. Wong receives research support through the University of California, Irvine from Amgen and Novartis and is a consultant and speaker for Novartis and consultant for Ionis. Dr. Hoogeveen has received research support through Baylor College of Medicine, Houston from Denka Seiken and is a consultant for Denka Seiken. Dr. Hu is an employee of Novartis Pharmaceuticals Corporation. The remaining authors have no relevant disclosures.
